# Neurocognition of dance: Guidelines for using dance as model behavior and research subject in cognitive neuroscience

**DOI:** 10.1016/j.isci.2026.117810

**Published:** 2026-11-02

**Authors:** Vasiliki Meletaki, Julia F. Christensen, Kohinoor M. Darda, Laura Rai, Andrea Orlandi

**Affiliations:** 1 Penn Center for Neuroaesthetics, University of Pennsylvania, Philadelphia, PA, USA; 2 Department of Cognitive Neuropsychology, Max Planck Institute for Empirical Aesthetics, Frankfurt am Main, Germany; 3 Advancement and Research in the Sciences and Arts (ARISA) Foundation, Pune, India; 4 School of Allied Health and Life Sciences, London South Bank University, 103 Borough Road, London SE1 0AA, UK; 5 Institute of Cognitive Neuroscience, University College London, Alexandra House, 17 Queen Square, London, UK; 6 Social Brain Sciences Lab, ETH Zürich, Zürich, Switzerland; 7 School of Psychological Sciences, Macquarie University, Sydney, NSW, Australia; 8 Department of Psychology, Sapienza University, Rome, Italy

**Keywords:** dance, neuroaesthetics, empirical aesthetics, dance expertise, research design, ecological validity, reproducibility, replicability, guidelines, wellbeing

## Abstract

Dance and movement science bridges cognitive psychology, neuroscience, cultural studies, philosophical aesthetics, and the performing arts. The field has evolved significantly, examining physical, social, psychological, and neural aspects of dance and movement execution and perception, contributing to a deeper understanding of human cognition and of dance itself. As the field expands, shared guidelines that support methodological rigor, reproducibility, and cultural relevance are of paramount importance. This paper synthesizes current research and introduces a comprehensive seven-principle research design framework for using dance in cognitive neuroscience. Key elements include the operationalization of “dance” in empirical sciences, the definition of who is a dancer, measures of expertise, and the importance of a culturally sensitive, holistic, and interdisciplinary approach to its investigation in the laboratory and in ecologically valid settings. This framework benefits research in dance and movement aesthetics, as well as investigations in social cognition, affective neuroscience, computer science, and artificial intelligence.

## Graphical abstract

**Figure:**
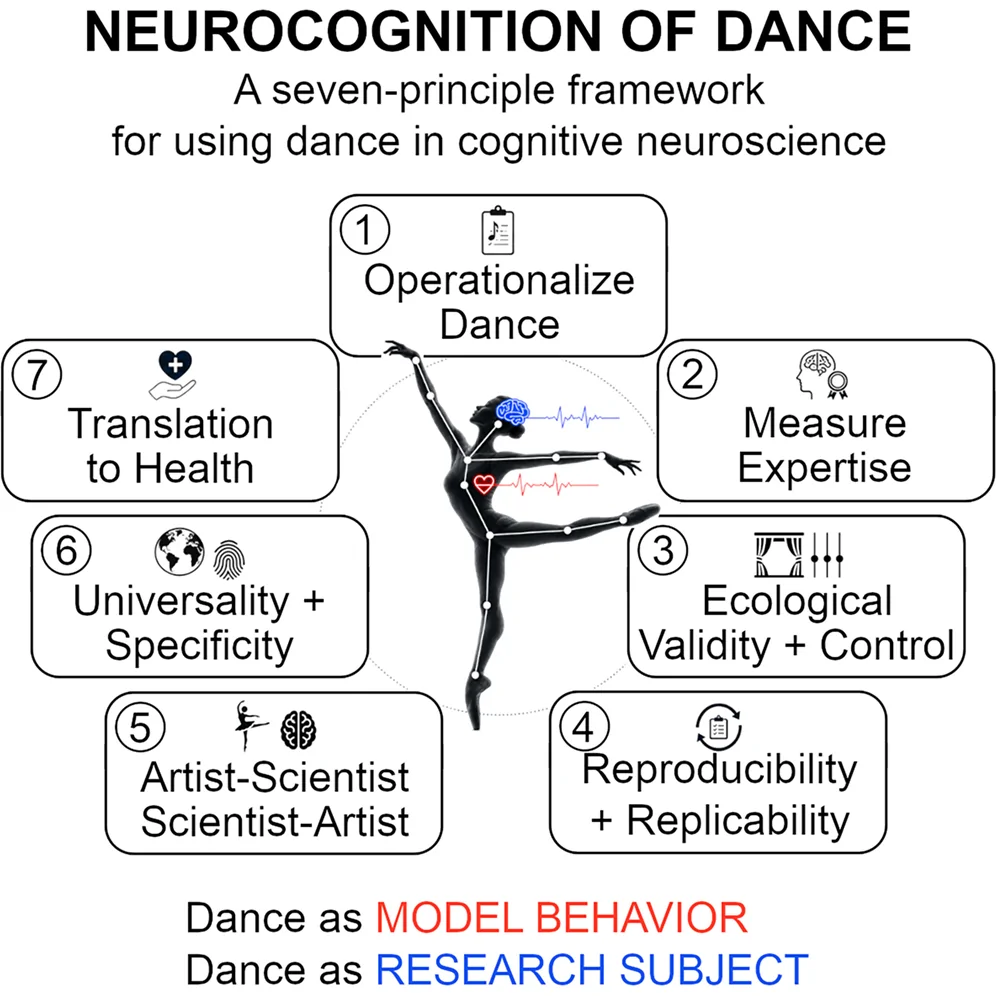

## Teaser

Natural sciences; Biological sciences; Neuroscience; Behavioral neuroscience; Social sciences; Psychology; Research methodology social sciences

## Introduction

Empirical research using dance as a model behavior to study neurocognitive processes, and dancers as neurocognitive models of expertise, offers valuable insights into how the human brain processes, learns, and responds to non-verbal, expressive, full-body movement and social interactions. Dance practices support cognitive rehabilitation and recovery from dysfunction, sometimes with higher success rates than traditional rehabilitation regimes.^1^ Evidence from the past 20 years supports the relevance of studying dance in basic and applied research across psychology, neuroscience, education, and computer science.^2^ However, guidelines and best methodological practices still need to be established to ensure the rigor, quality, and validity of such research,^3^ particularly given the methodological heterogeneity that may contribute to variability in reported outcomes.

Although dance is a complex phenomenon, it can be operationalized and investigated with the same scientific rigor applied to other forms of human movement. In July 2022, we, the authors of this paper, hosted a symposium titled *“Emerging Multi-disciplinary Perspectives and Cutting-Edge Advancement in Dance Neuroaesthetics”* at the ESCAN (European Society for Cognitive and Affective Neuroscience) biennial meeting. We highlighted state-of-the-art methodologies, challenges and open questions, and proposed a comprehensive seven-principle research design framework for using dance in cognitive neuroscience as a model behavior to investigate human cognition, or as a subject of research in itself. This paper is the result of the interdisciplinary dialogue between the authors that followed from this meeting. See Figure 1.

**Figure 1:**
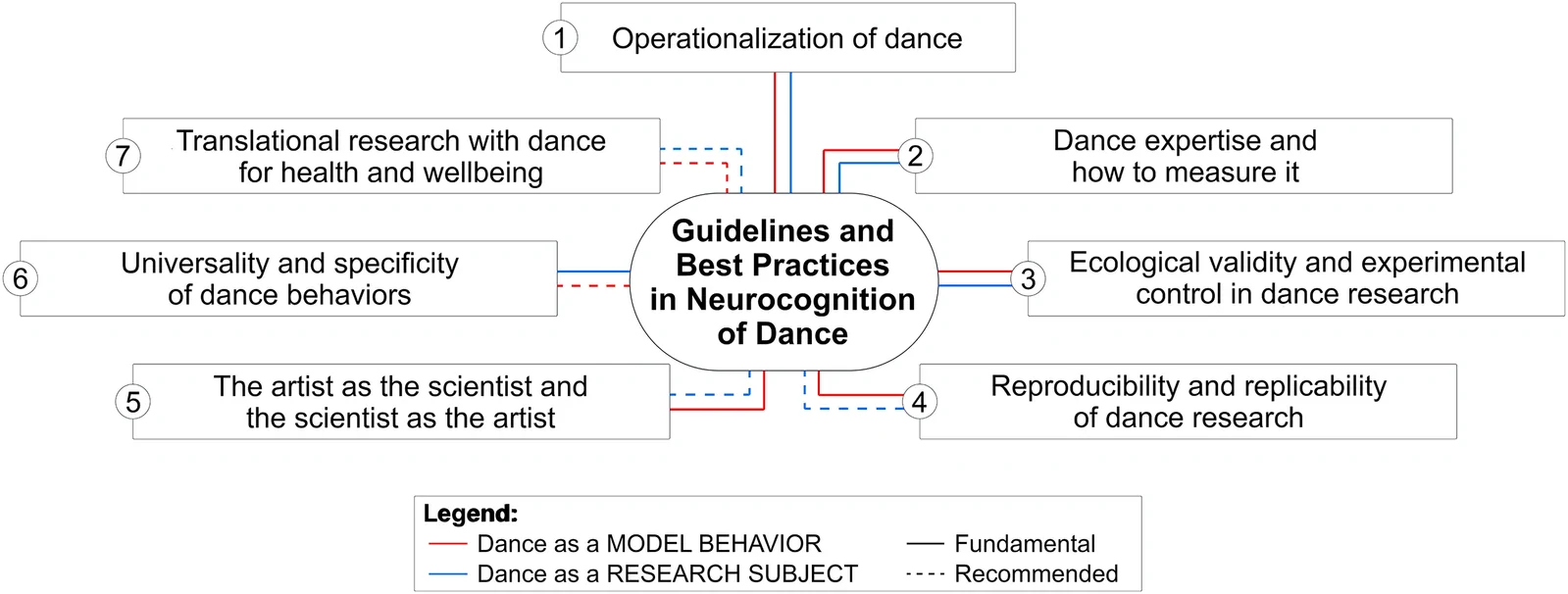
A seven-principle framework for refining research questions, designs and methods in neurocognition of dance This conceptual map highlights how, depending on the research focus (i.e., dance as a tool/model behavior or as a subject of research), specific principles may be fundamental for conducting rigorous, impactful, and reproducible studies in the field of human dance, while others are strongly recommended.

The manuscript offers a guide for operationalizing dance (Section operationalization of dance), defining and measuring dance expertise (Section dance expertise and how to measure it), maintaining ecological validity across laboratory and real-world settings (Section ecological validity and experimental control in dance research), improving reproducibility and methodological standards (Section reproducibility and replicability of dance research), fostering interdisciplinary approaches (Section the artist as the scientist and the scientist as the artist), incorporating cross-cultural and individual difference perspectives (Section universality and specificity of dance behaviors), and designing translational research in health and wellbeing contexts (Section translational research with dance for health and wellbeing). See Box 1.

Box 1 **Core questions and recommendations of the seven-principle framework**

**Table:** 

Principle	Core question	Best Practices and Recommendations
1. Operationalization of dance	What is “dance” in scientific research?	• provide a clear definition of what is meant by “dance” in a study. • provide a rationale for selecting a specific dance style/technique based on movement features of interest. • provide a rationale for selecting a specific dance style/technique, considering the specific neurocognitive mechanisms (e.g., entrainment, imitation, enculturation) and a clear research question. • avoid generalizing findings across dance styles or cultures without acknowledging the dance styles’ differences in structure, technique, aesthetics, cultural context, and embodied meaning. • especially in cross-cultural settings: Transparently justify and document ambiguous classifications and labeling of dance styles (e.g., in footnotes or providing additional references about nomenclature, history and documentation—see, for instance, note “c” in Table 1 in this article). • consult Tables 1 and 3 to align style/technique choice with research goals.
2. Dance expertise and how to measure it	Who qualifies as a “dance expert” in scientific research, and how can expertise be objectively assessed?	• use multidimensional definitions of expertise, going beyond years of training. • consider different dance activities or behaviors that possibly develop distinct neurocognitive competences: daily training, live performance, rehearsal, teaching, choreography, improvisation. • report and differentiate between visuomotor and visual expertise for different styles/techniques. • report the age of onset. • assess expertise using validated inventories (e.g., Gold-DSI), and complement them, where possible, with physiological or psychometric measures (e.g., BMI, interoception accuracy). • consult with dance experts (e.g., performers, teachers, choreographers) to ensure accurate classification of dance training and style.
3. Ecological validity and experimental control in dance research	How can researchers maintain experimental control while capturing the real-world complexity of dance?	• sometimes fully ecological means that experimental control is absent or nearly absent, limiting generalizability. • consider the limitations of traditional laboratory settings where possible. • explore the possibility of live/social settings for more ecologically valid studies. • consider multiple experiments, where results obtained in live settings are followed up with step-by-step replication of findings under controlled conditions. • select stimuli based on the setting (laboratory vs. live/social space) and research question (e.g., images, short videos, full performances, live events), aiming to balance experimental control and ecological realism. • combine behavioral data collection with techniques assessing brain and body responses (e.g., whether to prioritize spatial or temporal resolution), possibly through interdisciplinary collaborations to maximize available resources and equipment. • consider mobile brain/body imaging (e.g., wearable EEG, fNIRS) to measure neural activity in ecological settings when feasible. • explore two-person neuroscience approaches^4^ and/or neural synchrony (e.g., INS) to capture social dynamics between performers and spectators. • integrate tools and techniques from other fields, such as computer vision (e.g., OpenPose, DensePose), to increase control over variables of interest. • avoid reducing dance to quantifiable kinematic features alone; consider physical presence, set design, and temporal structure.
4. Reproducibility and replicability of dance research	How can empirical dance-based research align with rigorous and transparent scientific practices to enhance its reproducibility and replicability?	• provide transparent descriptions of stimuli, dance styles, research settings, participant inclusion criteria, and rationale for your choices (e.g., style/technique selection). • conduct *a priori* power analyses and report effect sizes to support statistical validity. • pre-register studies and clearly document methodological and analytical decisions. • use open and reproducible workflows (e.g., share data/code when possible) to enable comparison and replication. • subscribe to open science practices. • share stimulus sets with detailed metadata (e.g., movement vocabulary, contextual features, recording parameters).
5. The artist as the scientist and the scientist as the artist	How can meaningful collaborations between scientists and artists enhance dance research?	• build interdisciplinary teams that include scientists, dance professionals, and experts in different technological and artistic fields. • co-develop research questions and methods (e.g., stimulus design) leveraging both scientific and artistic knowledge. • explore embodied neuroscience (e.g., scientists engaging in movement tasks) and experimental choreography (e.g., artists designing performance as “scientific experiments”). • use motion capture tools and analysis frameworks (e.g., EMOKINE) to integrate artistic and scientific data. • promote interdisciplinarity as a means to support social cognition research, artistic innovation, and the application of embodied knowledge to health and education.
6. Universality and specificity of dance behaviors	How can researchers design studies that account for both the shared and culture-specific dimensions of dance?	• consider dance as both a culturally constructed and neurocognitively grounded behavior. • acknowledge that core mechanisms (e.g., entrainment, simulation) may be shared, but their expression and interpretation vary across cultural contexts. • assess participants’ cultural background, prior exposure, and enculturation/acculturation experiences when investigating dance observation or evaluation. • avoid generalizing from Western dance styles and training traditions to all other dance forms (and vice-versa). • given the over-focus on WEIRD dance in science, include non-WEIRD dance styles, traditions, and participants in research. • use mixed methods and cross-cultural comparisons to reveal both universals and specificities in cross-cultural dance perception.
7. Translational research with dance for health and wellbeing	How can dance-based interventions be optimized for clinical and societal applications?	• differentiate between general dance practice and dance movement therapy (DMT) when designing studies. • clearly describe the style(s) of dance used (e.g., partnered, group, traditional) and their therapeutic rationale. • ground dance intervention studies in core therapeutic mechanisms, and collaborate with a certified professional. • use standardized/validated outcome measures and report key intervention variables (e.g., frequency, duration, group size, facilitator credentials). • prioritize methodological rigor in clinical studies: larger sample sizes, randomized controlled designs (RCTs), blinding, and long-term follow-ups. • develop and test disorder-specific dance intervention protocols using DMT as a model of how to operationalize treatment mechanisms. • ensure inclusivity in sample composition by attending to gender, ethnicity, and representation of Indigenous and minoritized populations. • promote interdisciplinary collaboration between clinicians, therapists, dancers, and researchers.

*Note*: Core questions and summary of best practices and recommendations for implementation of the framework. In this table, “best case scenarios” are presented. Often, lack of funding forces dance-based scientists to make compromises, opening the door to criticism. This table is an invitation to mention these points in the discussion to ensure that best practices continue to be communicated.

Our methodological framework draws on empirical work, literature reviews, and meta-analyses demonstrating how dance provides:

(i) a model behavior for investigating the neural and cognitive mechanisms underlying action- and emotion perception, and movement representation.^5^^,^^6^^,^^7^ Research has established how dance observation recruits visuomotor (simulation) processes depending on the observer’s expertise, and how observers decode expressive whole-body movement in terms of affective and intentional meaning. For a review, see Foster Vander Elst et al. (2023).^6^
(ii) a tool to study the interplay of brain networks affording aesthetic appreciation of movement,^8^^,^^9^^,^^10^ such as the visual, sensorimotor, and reward systems. Studies in empirical aesthetics have determined how movement features, expertise, and contextual framing contribute to subjective evaluations and aesthetic experience of dance. For a review, see Kirsch et al. (2016)^9^ and Orlandi & Candidi (2025).^10^
(iii) a neurocognitive model of expertise to study neuroplasticity and learning.^11^^,^^12^^,^^13^^,^^14^ Comparisons between trained dancers and non-dancers have revealed training-related differences in brain structure and connectivity, providing a model for studying experience-dependent plasticity. For an overview, see Karpati et al. (2015).^13^
(iv) a valuable tool for rehabilitation and recovery from disease and dysfunction in clinical settings.^15^^,^^16^^,^^17^^,^^18^ Dance movement therapy and other dance-based interventions have been shown to have positive effects on motor, cognitive, and mental health outcomes across neurological and psychiatric populations. For a review, see Millman et al. (2021).^15^

A comprehensive discussion of this literature lies beyond the scope of this paper. Focusing instead on providing a methodological framework, this work contributes to improving theoretical precision, research questions, operationalization, and reproducibility in dance-based cognitive neuroscience and related fields.

### Operationalization of dance

Everyone recognizes dance, yet no two people’s notions of what dance is are exactly the same. The term “dance” refers to behaviors ranging from simple rhythmic groove movements performed to a regular beat, to codified dance styles that can be learned by anyone, to highly structured movement repertoires requiring years of athletic and artistic practice. In some cultures, dance is not even differentiated from other performing arts such as music, song, and percussion.^19^^,^^20^ See Table 1 for examples and key differences between dance styles in terms of neurocognitive predispositions involved and potentially modulated by dance. These are important for defining and operationalizing dance in empirical research.

**Table 1:** Key dimensions differentiating dance styles and techniques

No.	Dimension	Examples from the opposite ends of each dimension	
1	movement complexity: number and length of motor sequences and repertoire to be learned	dance styles with fewer, perhaps repetitive movement sequences (e.g., folk line dances)	dance styles with extensive movement vocabulary and structured repertoire (e.g., Bharatanatyam, Ballet)
2	music and rhythm: presence and type of musical structure	dance forms performed in silence or with unconventional soundscapes (e.g., Butoh, experimental contemporary dance)	dance styles performed to groove-based or rhythmic music (e.g., salsa), or with “classical” music of a culture
3	proximity and social touch: presence of one or more dancers in physical space	solo dances with minimal or no physical proximity to others (e.g., jazz, burlesque, tap dance, Zeibekiko)	partner, group, and other contact-based dances (e.g., ballroom, contact improvisation)
4	intention^a^: Performative vs. Personal; = the intention is ‘to compete or perform’ vs. to connect socially or with oneself	dance forms without a performative focus (e.g., some recreational dance communities; dance movement therapy)	styles performed in cyphers, shows, or competitions (e.g., breakdance battles, proscenium stage)
5	expression^b^: Formalist vs. Expressionist	formalist dance styles that focus on form, precision, and “the look”: Classical Western Ballet; Chinese Classical Dance; some Western partner dances; some Western Contemporary dance (e.g., Merce Cunningham, Lucinda Childs)	expressionist styles aim to convey emotion and inner states: Some Classical Western Ballet practices; some Western Contemporary dance (e.g., Pina Bausch, Gaga technique); Iranian Classical dance; Butoh; Bharatanatyam
6	narrative: presence vs. absence of a clear narrative	absence of clear story lines: Western partner dances; Neoclassical Western ballet (formalist styles; e.g., Balanchine); some Western Contemporary dance (e.g., Merce Cunningham, William Forsythe)	presence of clear story lines: African folk dances (e.g., Gota); classical Western Ballet; Bharatanatyam; some Western Contemporary dance (e.g., Pina Bausch)
7	setting: cultural or physical context of performance	informal, community-based or street settings (e.g., street dance, folk dance)	“Classical”^c^ dance styles often performed in formal theater settings (e.g., Western ballet)
8	synchrony and coordination: requirement for coordinated and/or synchronous movement between dancers	individualistic and expressive dance styles require minimal synchrony and coordination with other dancers (e.g., vogue fem)	considerable coordination and synchrony required in partner or group dances (e.g., line dance, swing, salsa, Argentine tango) N.B. some dance styles are “mirroring” (i.e., full synchrony), such as swing and line dancing, while others are “responsive”, meaning that the dancers do not do the same move but respond to each other. This requires coordination but not exact movement synchrony, as when one partner makes a move, and the other executes a different but coordinated response (e.g., ballroom, Argentine tango)
9	accessibility for untrained viewers: how easily the dance can be appreciated or understood without formal training	forms requiring cultural or technical familiarity for full appreciation (e.g., Kathakali, Western postmodern dance)	styles with overt rhythm, emotion, or visual appeal accessible to all (e.g., Bollywood, flash mobs, ballet, Bharatanatyam)
10	perceived social status: association with institutional or social prestige	dances associated with elite cultural institutions or classical tradition (e.g., Western ballet, Indian classical)	dances perceived as grassroots or alternative (e.g., folk, street, pole dance)
11	sociopolitical origin: influence of cultural and political context on the dance	commercialized or recreational dance styles with limited sociopolitical anchoring (e.g., Zumba, aerobics dance)	dance forms shaped by sociopolitical movements (e.g., Tahitian dance as cultural resistance during colonization)

*Note*: The left column shows 11 dimensions to consider during dance-style selection for an experiment due to differential neurocognitive mechanisms and processes engaged by the dance styles. The two columns to the right show examples from the opposite ends of each dimension. Refer to Figure 2 for a visual illustration with three examples of dance styles.

a The nature of a dance, whether social or competitive, can also depend on the dancer’s mindset or intention. For example, an Argentine tango milonga may serve purely as a relaxed, social gathering focused on internal connection (e.g., dancers moving with eyes closed), or as a highly performative and competitive display influenced by external attention (e.g., dancers hunting the gaze of others).

b Most dance styles contain expressive elements, but the extent and form of expressivity can vary widely. For instance, Bharatanatyam places strong emphasis on storytelling, with specific hand gestures, facial expressions, and eye movements, with clear meanings to the trained eye, choreographed into its structure. While Western ballet also has codified repertoires and expressive potential, recreational dancers may not engage with these narrative elements as deeply. Interestingly, both Bharatanatyam and ballet allow for complete improvisation, in which expressivity becomes more personal and may not follow a shared or recognizable code among practitioners.

c The term ‘classical’ dance is used here to refer to historically codified dance styles and techniques such as Western classical ballet and Indian classical dance (e.g., Bharatanatyam). These styles are traditionally performed in formal theatrical settings, typically featuring conventional staging, a proscenium layout, and a clear separation between performers and audience. More broadly, borrowing from scholarship on classical music and the arts, classical refers to cultural practices that are formalized through theory and pedagogy, shaped by specific social and historical contexts, and maintained across generations through institutional teaching, expert transmission, and a recognized canon of exemplary works.

In empirical research, scientists deconstruct phenomena into “independent variables” to study how they contribute to specific outcomes ("dependent variables"). Depending on the neurocognitive mechanism under investigation and the research question, one dance style may be more appropriate than another. Using the following four neurocognitive mechanisms, an example is provided for how research questions can be organized. (These four are illustrative and do not represent the full range of neurocognitive processes associated with dance. For a more extensive mapping of processes, see Section dance training, dance behaviors, and neurocognitive competences; Table 3).

(i) *Action simulation and entrainment* – enabling humans to *follow and synchronize their body movements* with external rhythms or movements of other agents.^7^^,^^21^^,^^22^^,^^23^
(ii) *Neural plasticity*/*motor skill learning* – reflecting adaptive (brain and body) changes associated with training, rehearsal, and repeated performance.^12^^,^^13^^,^^24^^,^^25^
(iii) *Imitation* – enabling *socialization* through the acquisition and enactment of shared movement practices (e.g., who dances, when, and how).^26^^,^^27^^,^^28^
(iv) *Enculturation* – influencing how dance is perceived, interpreted, and valued within culturally specific contexts.^29^^,^^30^^,^^31^

These mechanisms emerge early in life, while their expression and refinement depend on neurodevelopmental maturation, exposure, motor learning, and practice.^32^^,^^33^^,^^34^^,^^35^^,^^36^^,^^37^^,^^38^^,^^39^^,^^40^^,^^41^ The engagement of these four may vary considerably, for example, depending on whether participants within an experiment watch a performance of Western contemporary dance or Indian classical Bharatanatyam.

(A) A Western contemporary dance performance, unfamiliar to anyone watching. Two dancers walk on stage, make eye contact with the audience, and grin manically while standing still, without music, external rhythm, or synchronized movements.^42^^,^^43^^,^^44^^,^^45^ In this performance, non-linear narratives, improvisation, and immersion are core aesthetic features.^20^^,^^46^
(B) An Indian classical Bharatanatyam performance, where a dancer moves to familiar, mostly rhythmic music and conveys popular mythological stories, especially to those familiar with the mythology of that culture. The aesthetic features here are codified hand and facial gestures (mudras), body postures, and full-body movements that “tell” a story, in addition to moments of synchronous movement.^47^^,^^48^

These two dances also differ with regard to other dimensions among the 11 outlined in Table 1. Figure 2 compares these two dance styles with a third with respect to these dimensions. Even within the same dance style, dimensions such as co-presence, social touch, and synchrony may vary across performances, possibly engaging different neurocognitive processes in performers and observers.

**Figure 2:**
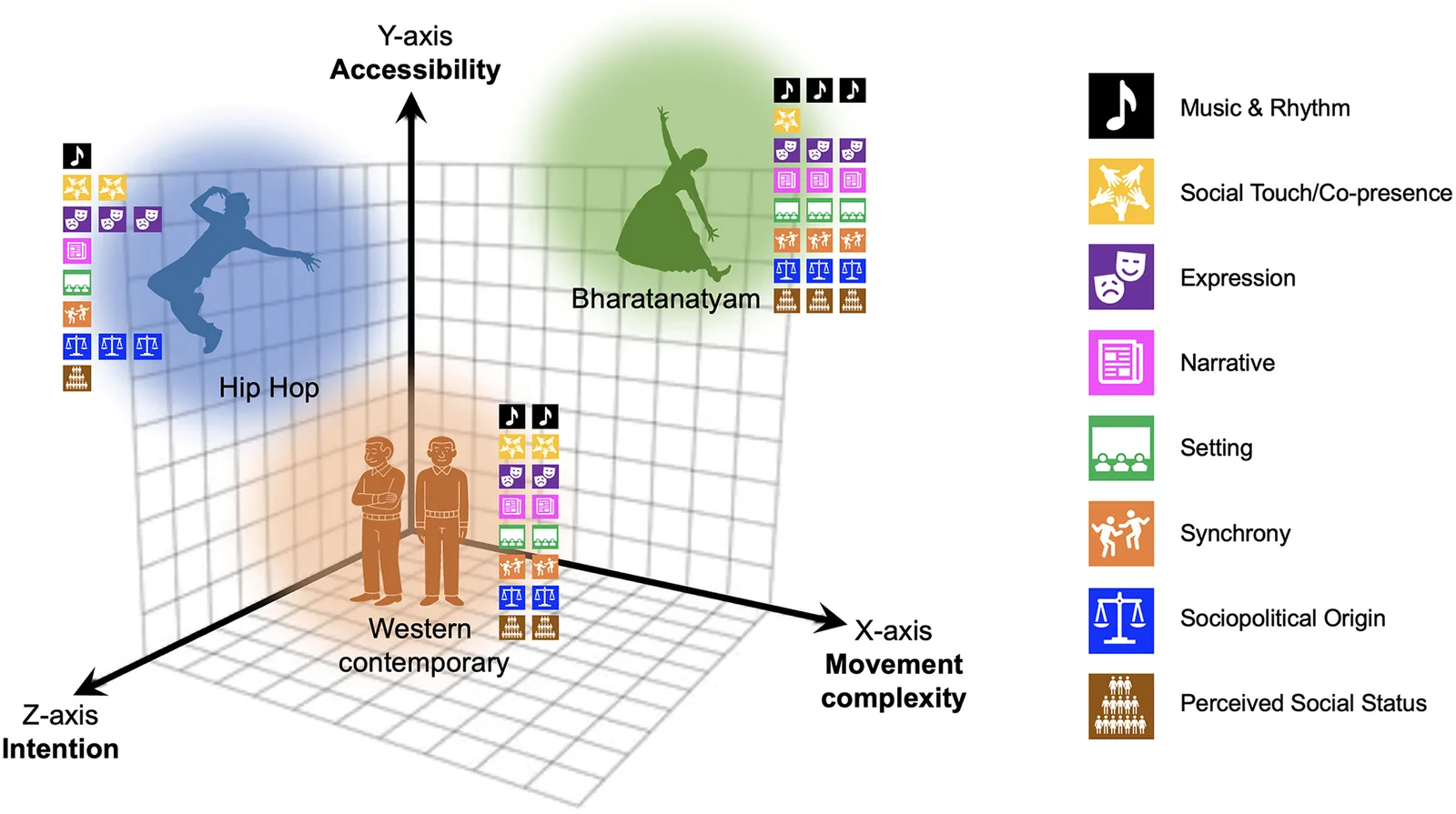
Classifying dance styles worldwide according to 11 dimensions Dance styles differ according to 11 dimensions that are relevant to empirical research with dance because these differences likely result in the engagement of different neurocognitive processes, such as (i) action simulation and entrainment, (ii) neural plasticity/skill learning, (iii) imitation and socialization mechanisms, and (iv) enculturation mechanisms. X axis: Movement complexity (few movement sequences – extensive movement sequences). Y axis: Accessibility for untrained spectators (not accessible – very accessible). z axis: Performative intention (no performative focus – performative/competitive focus). Icons symbolize the other eight dimensions. One icon versus three icons indicates the extremes of the continuum; two icons designate the middle. Two performances of the same dance style may differ in this classification, and dance professionals may need to discuss and agree on these classifications for each instance (e.g., in terms of how much social touch/co-presence or synchrony there is). Please refer to Table 1 for extensive descriptions of each dimension and its continua. Such classification of the dance style used in a study is recommended prior to conducting the research. Copyrights: Icons by Microsoft Word. Free dance vectors by Vecteezy (www.vecteezy.com/free-vector/dance); Indian and hip-hop dancer by K for Kreative; contemporary dancers by Mubashir Sayyam Khan. Composition: JFC & Felix Bernoully.

Conclusions drawn from studies using these performances would be problematic without a clear conceptualization of what is meant by “dance” in each context and of the neurocognitive process under investigation. From a neuroscience perspective, the two examples represent very different classes of behavior.

#### Best practices and recommendations

Define what is meant by “dance” in the context of your study. Classify dance behaviors according to the neurocognitive mechanisms needed to perform and perceive them (refer to Table 1; Figure 1; see also Table 3 in section dance training, dance behaviors, and neurocognitive competences). Justify the selection of a specific dance style or technique based on movement features, dance dimensions of interest (e.g., movement complexity, music and rhythm, proximity and social touch, intention, expression), research questions, and neurocognitive mechanisms of interest (e.g., entrainment, plasticity/expertise, imitation, enculturation). Avoid generalizing findings across dance styles, techniques, or cultures without acknowledging these differences and discussing their implications. Ambiguities in classification are often part of cross-cultural research and should be addressed transparently, with decisions justified and documented, for example, through footnotes or supplementary material (e.g., ritual movement practice operationalized as “dance” in research on rhythmic coordination).

### Dance expertise and how to measure it

Central to the question of what *dance* is*,* is *who* is considered a *dancer*. Expertise studies in cognitive neuroscience require operationalizing the level of skill. The Cambridge Dictionary^49^ defines *expertise* as “a high level of knowledge or skill”, but a precise and standardized quantification of what constitutes a “high level” remains elusive in cognitive neuroscience and psychology, particularly in sports and the performing arts.^50^^,^^51^

Formal dance training changes the body, brain structure, and function.^13^ It provides complex technical visuo-motor skills to perceive, reproduce,^52^ and deconstruct complex movement into its constituent movement chunks.^53^ Watching dance regularly also modulates visual, motor, and memory systems,^9^^,^^54^^,^^55^ contributing to what is summarized as “visual dance expertise”.

Depending on expertise level and professional status, dance training includes: academic technique training, expressive practice, improvisation, partnering work, group synchronization practices, repertoire practice, musical practice, stage rehearsals, live performances, teaching, and choreography. These activities refine somatosensory and motor processes, memory, attention, emotional processes, non-verbal communication, social cognition, and higher-order skills such as divergent thinking (see Section dance training, dance behaviors, and neurocognitive competences).

A number of questionnaires have been proposed to measure expertise across these domains. An overview of standardized inventories is provided in Table 2. Most have only recently been made publicly available^56^ or are not yet published in peer-reviewed journals.^58^ They were primarily developed in Western dance contexts and do not yet capture all dimensions of dance engagement, especially informal or digitally mediated participation^62^). Existing questionnaires should therefore be considered useful tools for characterizing dance expertise rather than definitive standards for all dance populations. Future research should develop and validate measures capable of capturing emerging forms of dance engagement and training, including those enabled by rapidly evolving digital and immersive technologies.

**Table 2:** Measures of dance expertise

Measure	What does it measure?	Benefits	Limitations
Goldsmiths Dance Sophistication Index (Gold-DSI)^56^	• visual, motor, and conceptual expertise • participatory and observational dance experience • subscales: body awareness, social dancing, urge to dance, dance training	• very comprehensive assessment of visual, motor, and conceptual expertise and expression • applicable for amateur and social dancers • includes often-overlooked typical behavior of dance enthusiasts: watching dance videos on the internet;	• limited information on professional expertise • no distinction between dance professions • assesses age in age-brackets • years of dance experience are reported within a range of years
Montreal Dance and Music History Questionnaire (MDMHQ)^57^	• includes total years of dance experience, principal style, other dance styles, music experience • focus on self-reported motor training	• covers the most reported general information on expertise (total years of expertise, principal style)	• focus on motor component • not publicly available • no distinction between dance professions
Dance Aesthetic Fluency Scale (DAFS)^58^	• focus on conceptual expertise and aesthetic appreciation • familiarity ratings on 14 items about dance terminology and history	• appears to be the only in-depth measure of conceptual knowledge and aesthetic appreciation • distinction between dance professions (professional dancers, hobby dancers, dance scholars, or dance critics)	• focus only on classical ballet and Western contemporary • focus on conceptual and aesthetic elements only • self-reported familiarity • not publicly available
Performance Competence Evaluation Measure (PCEM)^59^	• qualitative assessment of a dancer’s proficiency in full-body involvement, body integration and connectedness in movement, joint articulation, movement skills.	• the only measure with external judges and scoring criteria • the only assessment of performed movements on the spot • suitable for all expertise levels	• time and resource intensive • focus on motor aspects only • focus only on classical western ballet and contemporary movements. • not validated
High Intensity Dance Performance Fitness Test^60^	• physical assessment of aerobic fitness (measured by HR changes) • adapted to contemporary dance and intermittent pattern.	• the only physical assessment of physical fitness adapted to dance • suitable for all expertise levels	• time and resource intensive • focus on motor aspect • focused on Western contemporary dance movements • not validated
Creative Achievement Questionnaire (CAQ)^61^	• self-report measure of achievement across 10 domains of creativity	• short (only 8 items) • suitable for all dance expertise levels • expertise assessment based on achievement in the domain of dance (e.g., I have danced on stage and been paid for it, I have been featured in international news outlets for my performances, and so forth)	• dance is only a sub-section • limited information on expertise • somewhat arbitrary questions to determine achievement in dance

There is not yet a unified measure that combines practice-oriented and achievement/skill-oriented measures of dance expertise. A recent genetically informative study of 6610 Swedish twins suggests that factors contributing to dance achievement may not fully overlap with those associated with engagement and practice levels, highlighting the importance of considering both dimensions when characterizing dance expertise.^63^

The most common practice in published experiments involving dance “experts” has been to report years of dance practice. Under this measure, “expertise” broadly includes all time spent in dance-related activities. Therefore, what constitutes “an expert” varies greatly across studies, from 3 years^64^ to 18 years of practice.^65^ Such discrepancies complicate cross-study comparisons. In what follows, the daily training behaviors relevant to dance expertise measures are outlined.

#### Dance training, dance behaviors, and neurocognitive competences

In *daily academic training*, dancers learn technically complex and precise movements, often in coordination and synchronization with others, and usually to music. Training builds stamina, endurance, breathing control, and memory for long movement sequences. It enhances proprioception, interoception, and the integration of visual, motor, and auditory information, which support action anticipation, error detection, motor control, motor learning, interpersonal coordination, and memory.

Through *live performances*, dancers develop *stage presence* and the ability to captivate an audience through their expressivity.^66^^,^^67^ They refine non-verbal communication and learn to convey emotions and meaning through movement. A broader movement repertoire enhances the range and precision of bodily expressions,^68^ supporting emotion expression and recognition, emotion regulation, self-awareness, and multisensory sensitivity to audience feedback.^69^^,^^70^ Performance also requires memory recall, interpersonal coordination, multisensory integration, and management of attention across dancers, the stage environment, and the audience.

*Rehearsals* are crucial for refining choreography, movement execution, and expressive skills.^71^ Dancers combine physical and mental practice,^72^ alternating between reproducing movement sequences and mentally simulating them.^73^^,^^74^ Kinesthetic (or motor) imagery is a powerful training tool in dance and other physical disciplines,^75^ supporting multisensory integration and enhancing body awareness.^76^ Depending on dance style, dancers may engage in auditory imagery, involving mental representations of music and sounds, as well as interoceptive or somatic imagery, involving representations of internal bodily states.

Three additional behaviors warrant attention when defining expertise: *teaching,*^39^^,^^77^ *choreography,*^78^ and *improvisation.*^79^ *Teaching* movement to others strengthens verbal and non-verbal communication and fosters social cognition, including empathy and theory of mind.^39^ These processes are supported by attentional focus and by the ability to create and structure motor chunks, both critical for effective knowledge transfer.^80^^,^^81^

*Choreography* demands advanced visuospatial and memory skills, higher-order functions such as creativity (convergent and divergent thinking), and decision-making. These cognitive abilities enable dancers to design pieces that are emotionally expressive and meaningful (e.g., dramaturgy), visually engaging, novel, and captivating.

*Improvisation* is an integral part of a dancer’s skillset.^79^^,^^82^ It emerges from kinesthetic experience, movement habits, physical constraints, training, and memory, while employing divergent thinking during decision-making.^82^ Although central to Western contemporary dance,^83^^,^^84^ contact improvisation, breakdancing, and Iranian classical dance,^29^ it is relatively uncommon in codified or highly structured styles such as ballet and modern dance (e.g., Graham, Horton, and Cunningham), which emphasize mastery of set vocabulary and technique.^85^^,^^86^^,^^87^ Dance styles vary in the extent to which they incorporate improvisation, and future research may benefit from considering improvisational practice in assessment of dance expertise. Compared to daily training, improvisation involves quickly adapting to environmental changes (e.g., sounds, other dancers) and engages divergent thinking, decision-making, multisensory integration, and interpersonal coordination.

For instance, contact improvisation differs from Western classical ballet not only in its reliance on improvisation, but also in the sensory modalities it emphasizes. While contact improvisation prioritizes touch, body awareness, and kinesthetic connection between dancers, ballet depends more heavily on visual information.^88^ This example shows why the neurocognitive competences developed through different dance behaviors cannot be fully captured by measuring “years of training” alone.

Dance experts also differ in the extent to which different neurocognitive competences are developed, depending on their level of engagement and the style or technique they practice. Table 3 outlines a practice-oriented and aspirational mapping of dance behaviors and the neurocognitive processes that may be associated with them. The table is intended to guide participant characterization and hypothesis generation, rather than to represent an exhaustive taxonomy or definitive evidence of one-to-one relationships between dance behaviors and neurocognitive processes.

**Table 3:** Assessing dance expertise: Neurocognitive processes likely stimulated by the practice of specific dance behaviors

		DANCE behaviors					
		Academic daily training	Rehearsals	Live performance	Teaching	Choreography	Improvisation
NEUROCOGNITIVE PROCESSES	**Perception**						
	proprioception	X	X	X			X
	interoception	X	X	X			X
	action anticipation/error detection	X	X	X	X	X	X
	entrainment/movement-music synchronization	X	X	X			X
	multisensory integration	X	X	X			X
	**Motor functions**						
	motor control/coordination	X	X	X			X
	motor learning	X					
	motor chunk creation				X	X	X
	interpersonal coordination		X	X			X
	**Memory**						
	memory encoding	X				X	X
	memory recall	X	X	X	X	X	X
	**Imagery/mental training**						
	(visual, motor, somatosensory, auditory)	X	X		X	X	X
	**Attention**						
	joint action/joint attention		X	X	X		X
	divided attention		X	X	X		X
	**Emotion**						
	emotion expression		X	X		X	X
	emotion regulation			X			
	emotion recognition			X			
	self-awareness	X	X	X			X
	awareness of audience reactions			X		X	X
	**Communication/Language**						
	non-verbal communication		X	X	X	X	X
	verbal communication				X		
	**Social cognition**						
	theory of mind			X	X	X	
	empathy		X	X	X	X	X
	**High-order functions**						
	convergent thinking					X	X
	divergent thinking					X	X
	decision making					X	X

#### Dance techniques, age of onset, visual and motor expertise, and multisensory training

Most formal dance education combines multiple *techniques* and somatic practices,^89^^,^^90^^,^^91^ making it difficult to identify dancers trained exclusively in one style. Consultation with a dance expert, such as a performer, teacher, or choreographer, is therefore strongly recommended when selecting participants across training backgrounds (see also Section the artist as the scientist and the scientist as the artist).

*Age of onset* refers to the age at which a skill is first acquired.^92^ Although no longitudinal studies have investigated the effect of long-term dance practice in children, short-term intervention studies suggest that dance can support the development of motor and executive functions, and social skills.^93^^,^^94^ Therefore, it is valuable to collect the age of dance training onset alongside existing expertise inventories (e.g., Gold-DSI^56^), which capture duration and extent of training but not when training begins.

Differentiating between *visuomotor* and *visual expertise* in dance is important because visual experience alone can lead to covert movement simulation, and the left premotor cortex may be sensitive to multisensory integration.^95^ However, there is no clear consensus on how best to quantify visual expertise in dance observers. Most studies rely on the number of live performances attended annually,^48^^,^^56^^,^^96^ while other forms of exposure, such as films (e.g., Bollywood), online videos, and social media, are rarely considered. These digital experiences often involve simple, repetitive routines that differ from professional or live performances,^96^ and it remains unclear whether they constitute meaningful visual expertise. Similarly, the influence of viewing medium, such as smartphone vs. large display, on biological motion perception has yet to be systematically examined. A promising development is the inclusion of dedicated items assessing observational experience in recent psychometric instruments, such as in the Gold-DSI.^56^

Dancers are not only trained in motor skills but also in artistic and *emotional expressivity*. Feelings, meanings, and narratives are conveyed through bodily movements. As a result, dancers may exhibit enhanced interoceptive accuracy or awareness. Moreover, expert dancers generally show superior physical fitness, lower body mass index (BMI), enhanced *proprioceptive abilities*, and greater *body awareness* than non-dancers.^97^^,^^98^^,^^99^^,^^100^^,^^101^ These characteristics are difficult to assess through self-report questionnaires alone, and the most appropriate measures of expertise may vary with the research question.

#### Best practices and recommendations

We propose defining dance expertise multidimensionally, considering activities such as daily training, live performance, rehearsal, teaching, choreography, and improvisation, that develop distinct neurocognitive competences (see Table 3). We recommend differentiating visuomotor and visual expertise across dance styles, reporting age of onset, and using validated inventories such as the Gold-DSI as part of a multidimensional assessment of expertise, ideally complemented by physiological and other psychometric measures relevant to dance training. Collaborating with dance professionals is essential for accurate classification.

### Ecological validity and experimental control in dance research

Most dance-related studies have been conducted in laboratory settings, with low ecological validity, where participants viewed dance videos or pictures, often in greyscale, with contextual elements blurred or removed. Figure 3 provides an overview of research settings, methods, and stimuli employed in dance research with regard to (1) the setting in which dance is performed and/or experienced and (2) the quality and realism of stimuli used to represent it.

**Figure 3:**
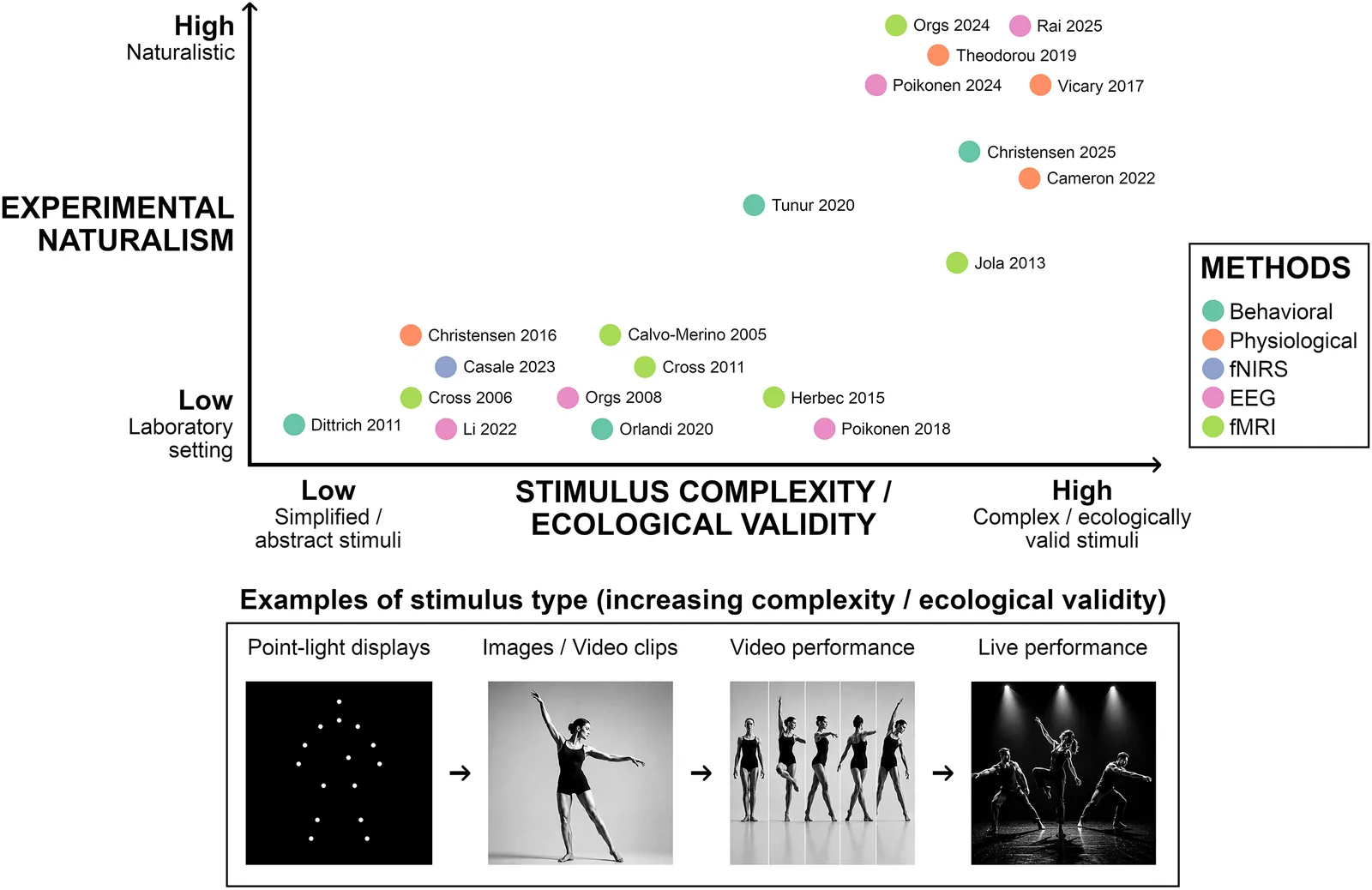
Experimental control and ecological validity in neurocognitive dance research Common stimulus types/complexity or ecological validity (X axis), experimental naturalism or research environments (Y axis), and research methods (bullet color) used in neurocognitive dance research.^7^^,^^21^^,^^23^^,^^45^^,^^70^^,^^102^^,^^103^^,^^104^^,^^105^^,^^106^^,^^107^^,^^108^^,^^109^^,^^110^^,^^111^^,^^112^

#### Experimental setting

Movement degrades signal quality in neuroimaging. However, advances in mobile brain imaging make it possible to record neural activity and cognitive responses in ecological (performance) settings,^113^ including real-world full-body behaviors,^114^^,^^115^^,^^116^^,^^117^ and dance^118^—on stage, in nightclubs, and in social spaces.

Dynamic social interactions can be indexed by measuring interpersonal neural synchrony (INS), the similarity in brain activity between individuals over time.^119^^,^^120^^,^^121^^,^^122^ However, most studies measure neural synchrony between individuals experiencing the same stimulus sequentially, rather than simultaneously, and studies involving large groups sharing the same physical space remain rare. One notable exception was Rai et al. (2025).^45^ An interdisciplinary team recorded mobile EEG from 23 audience members simultaneously in a theater. INS between spectators was highest in the delta frequency band and varied with the dancers’ movements and subjective ratings of expected audience engagement, as predicted by the choreographer and performer. Neural synchrony was highest during moments of direct interaction with the dancers (i.e., eye contact), and was significantly higher than during solo laboratory viewing of the same performance. These findings provide initial neuroscientific evidence that experiencing dance involves active communication between performers and spectators, and that artistic intentions can be “detected” as INS among audience members. Together with other studies,^119^^,^^123^^,^^124^ this illustrates the importance of co-presence for the full aesthetic experience, which is only possible in ecological settings.

While artifact rejection methods have been developed for cleaning movement-induced noise in EEG recordings, these have been evaluated primarily during walking or cycling, with limited testing for more complex, dance-like movements.^125^ There is, so far, no standard method for processing locomotive EEG data. It is therefore important to consider whether the type and range of movement involved in a choreography can be reliably measured using mobile brain imaging equipment. The mobile brain/body imaging approach^126^ combines physiological measurements, motion tracking, and data-driven EEG analyses to differentiate neural from non-neural activity. First studies have successfully implemented this approach, recording EEG in participants spontaneously moving to music.^127^

Methods from vision science can also support the study of cognitive processes in ecologically valid dance research. Eye-tracking can monitor observers’ visual behavior in laboratory and real-world environments. Although still limited, available evidence suggests that dance expertise enhances visual processing speed, potentially enabling experts to anticipate movements and process choreographic material faster. Expert dancers exhibit faster saccades and more clustered fixations, particularly when music and dance are synchronized.^128^^,^^129^ Measuring audience gaze with mobile eye tracking, especially when combined with objective estimates of body kinematics, may provide novel insights into collective attention during live dance performances (Arthuis et al., 2024. Conference poster at https://osf.io/4v7j2) and assist with eye-gaze/head movement artifact removal.

Combining methodological approaches, research beyond traditional laboratory environments can offer a more comprehensive understanding of the neural and cognitive processes engaged during dance performance and observation. Novel methods for artifact removal also support research in countries where access to traditional laboratory infrastructure is limited. However, efforts to increase ecological validity entail practical challenges and costs (challenging particularly for researchers working in under-resourced contexts). Besides, participants experience fatigue from wearable devices or extended recording time. These constraints may represent important challenges still to be resolved.

#### Dance as a “controlled” experimental stimulus

Early studies using dance as a research tool investigated neurocognitive functions such as action perception, visual processing, visuo-motor integration, and motor learning. These studies often employed videos of short dance sequences and recruited dancers as movement experts.^21^^,^^23^^,^^48^^,^^55^^,^^130^ Stimuli were typically brief (e.g., 5–20 s)^112^ and often drawn from Western classical ballet due to its formalization and experimental controllability. To maintain control, dance stimuli were often deprived of contextual information, with dancers filmed against plain backgrounds^31^ or reduced to point-light displays via motion capture.^131^ The advantage of such well-controlled paradigms is the ability to investigate the contribution of carefully controlled variables including temporal features (e.g., velocity, acceleration, smoothness, or synchrony), or spatial features (e.g., body positioning, visual overlap, or two-body entanglement^132^) to dance evaluation.^10^ Dance movement stimuli can be validated, either by comparing ratings from expert and non-expert dancers or by quantifying movement features. Algorithms based on deep neural networks, such as OpenPose^133^ and DensePose, can estimate body kinematics for statistical analyses.

Importantly, approaching dance purely as a set of reducible, quantifiable features risks overlooking the essence of the experience itself.^134^^,^^135^ Critical features are lost, including the performer’s physical presence and expressivity, set and lighting design, and the dynamic structure of choreography or narrative over time. Future research may address these limitations by leveraging technological advances in brain imaging and applying data-driven, multivariate time-series analyses to capture the multidimensional, embodied nature of dance as it is experienced in real-life settings.

#### Best practices and recommendations

Different research questions require different research settings. Traditional laboratory settings and ecologically valid, live, or social contexts each have specific advantages and limitations. When feasible, combining methods can provide converging evidence. Mobile brain/body imaging, including wearable EEG or fNIRS, allows neural responses to be collected in naturalistic settings. Combining qualitative dancers’ reports of movement intention with interpersonal neural synchrony (INS) and computer vision tools (e.g., OpenPose, DensePose) helps connect performer intention to observer perception without reducing dance to kinematic variables alone. Striking a balance between experimental control and ecological realism is key for meaningful generalization.

### Reproducibility and replicability of dance research

Amid growing scrutiny of research reliability, particularly in light of the replication crisis in psychology and neuroscience, dance research stands to benefit from adopting rigorous methodological and reporting standards.^136^^,^^137^^,^^138^^,^^139^^,^^140^ Given the variability described in sections operationalization of dance, dance expertise and how to measure it, and ecological validity and experimental control in dance research, detailed information on dance style choice, (1) participants’ characteristics (e.g., multidimensional assessment of dance expertise; Table 2) and (2) stimulus characteristics (Table 1; Figures 2 and 3) is required to allow evaluation, comparability, and replicability. Besides, Open Science practices ensure transparency, reproducibility, and interdisciplinary impact. These include open-access publications or preprints, open datasets, shared stimulus sets, protocols, and analysis code.

Dance research poses distinct challenges for reproducibility because of the embodied character, stylistic diversity, and cultural specificity of dance itself, as discussed further in Section universality and specificity of dance behaviors. Where feasible, sharing movement recordings or kinematic datasets in standardized formats can enable comparison and reanalysis. Because stimulus standardization is closely linked to ecological validity, shared stimuli should be supported by comprehensive metadata. Relevant descriptors include the number of dancers, movement vocabulary, or step names (where applicable; some dance techniques rely on formally defined vocabularies (e.g., classical ballet), while others do not), music and sound properties, costumes, lighting, stage design, and recording conditions. Visibility is also important: while open-access publications can increase access for academic and non-academic audiences, dissemination through press releases, podcasts, workshops, and public engagement initiatives raises the profile of this field. Besides, events that integrate scientific presentations with movement-based activities also foster dialogue between researchers, artists, and practitioners.

#### Best practices and recommendations

Provide transparent descriptions of stimuli and clear rationales for choosing specific dance styles. Specify participants’ inclusion criteria, favoring a multivariate assessment of dance expertise when engaging with professional or semi-professional dancers. Clearly justify research settings and design choices, especially in out-of-the-lab contexts. Conduct *a priori* power analyses, report effect sizes, and pre-register study protocols and analysis plans. Adopt open and reproducible workflows, sharing stimulus sets, data, and code where possible, to support replication, comparability, and alignment with broader Open Science standards.

### The artist as the scientist and the scientist as the artist

Science aims to systematize the natural, social, and personal phenomena into measurable variables that can be studied with qualitative or quantitative methods. Because methods differ across disciplines, interdisciplinary teams are essential, also in dance research. Just as meaningful scientific insights into athletic performance require collaboration with athletes, dance research needs collaboration with dancers, performers, and choreographers. Dance expertise alone is not sufficient to design scientifically rigorous studies, but plays a critical role in “translating” what dance is to non-dancer scientists within the team, enriching both the creative and the scientific process.^29^^,^^45^ Jola (2020)^141^ proposed two complementary approaches: (1) *embodied neuroscience*, in which scientists embody the physical practice of their topic of investigation, and (2) *experimental choreography*, in which artists engage with science so that performances emphasize both artistic and scientific value.^45^^,^^142^

Collaboration with artists provides guidance in navigating the diversity of dance styles, techniques, and training domains discussed in Sections operationalization of dance, dance expertise and how to measure it, and ecological validity and experimental control in dance research. Dance experts contribute to the creation and selection of movement features, ranging from single steps to complex sequences. Their knowledge of semiotics and dramaturgy (*semiotics* = how bodily gestures, postures, and spatial dynamics convey meaning, emotion, and narrative; *dramaturgy* = the conceptual, structural, and interpretive development of a dance piece) offers valuable insight for studying non-verbal communication through movement and helps overcome technical limitations, since some dance movements may be difficult to record using video or motion tracking suits, or may not be physically feasible under experimental constraints.^143^^,^^144^ Finally, interdisciplinary teams benefit from establishing shared terminology and definitions (for example, two individuals may be differently classified as “interacting” depending on whether the framework comes from social cognition or choreographic theory).

Recent interdisciplinary projects combining dance, digital humanities, computer science, psychology, and neuroscience have contributed to cultural preservation, technological innovation, and artistic practice, besides scientific advancement. Using a combination of filmography and motion tracking methods, for example, the Motion Bank project (https://www.motionbank.org/), the Virtual Dance Museum (https://dancemuseum.cs.ucy.ac.cy) for Cypriot folk dances,^145^ and the Iranian classical dance documentation project^29^^,^^31^^,^^146^^,^^147^^,^^148^ have contributed to dance documentation, transmission, and preservation. A methodological framework developed to aid such endeavors in the future is the EMOKINE framework, an open-access motion capture analysis tool that extracts statistics from kinematic features of full-body movement and is compatible with multiple motion capture systems.^149^

Expanding dance research into computer science and robotics has already borne fruit. AI-powered digital technologies^150^^,^^151^ and avatar-based media feed into the emerging area of “choreorobotics”,^152^ which applies principles of choreography, movement analysis, and somatic practice to expressive robotic systems. Besides, the novel technologies can inform choreographers about how aesthetic responses vary when dance movements are performed by a human dancer, a humanoid avatar, or an artificial agent,^142^^,^^153^^,^^154^^,^^155^ with results suggesting that, despite the seductive allure of the novel technologies, *human* dance remains the most aesthetically pleasing to the human brain. These developments also extend to dance production and educational contexts, where AI-based systems are increasingly explored as tools for creative collaboration (Jobin, G. (2024). CIE Gilles Jobin Studios44/MocapLab https://www.gillesjobin.com/en/)^,^^156^ and classroom-based movement interventions.^157^^,^^158^^,^^159^

#### Best practices and recommendations

Build interdisciplinary teams involving scientists, dance professionals, and experts from relevant artistic and technological fields as equal collaborators. Co-develop research questions, methods, and stimulus design, drawing on both scientific and artistic expertise. Where available, use validated tools (e.g., motion capture) and validated analysis software (e.g., EMOKINE), to support innovation in both the arts and sciences, and translate embodied knowledge into applications in education, society, and healthcare.

### Universality and specificity of dance behaviors

*Cross-cultural* empirical (neuro)aesthetics investigates the universality and distinctiveness of cognitive and neural processes underlying aesthetic experiences across cultures.^160^ Despite its ubiquitous presence across societies, dance remains a relatively neglected topic in this domain. In most cultures, dance occupies an important social role, while in others it is deeply embedded in religious, spiritual, or cultural practices. Cultures have developed unique dance traditions, each practiced for specific expressive reasons.^20^^,^^161^ Dance also fulfills multiple psychobiological functions and regulates social and emotional behaviors.^97^^,^^162^^,^^163^

Dance is culturally and socially constructed, yet it relies on core neurocognitive mechanisms such as motor control, entrainment, simulation, and expression. The multicultural nature of dance styles makes it difficult to define truly universal perceptual, cognitive, and emotional processes associated with “dance”.^164^ This cultural complexity must be accounted for in study design.^165^ However, many current investigations are limited by a broadly *Western*-centric perspective.^166^ Recognizing these cultural differences is crucial to understanding dance as a multisensory, multicomponent practice rooted in social and cultural contexts.

In immigration psychology, enculturation refers to the neurodevelopmental process through which individuals adopt cultural norms, while acculturation occurs when exposure to a new culture after migration shapes behaviors and preferences.^167^^,^^168^ These processes influence cultural life, including dance, and are shaped by both conscious and unconscious experiences.^169^ Despite their relevance, few cross-cultural studies have directly investigated differences and universals in dance perception and practice.

Recent studies demonstrate that dance evaluation is shaped by prior exposure, cultural background, enculturation, acculturation, and dance training, influencing emotional recognition and aesthetic preferences. Cross-cultural studies suggest that participants may show ethnocentric biases, meaning that they may evaluate or interpret dance more favorably when it is culturally familiar, as shown by Darda and Cross (2022),^170^ who also found that this bias was reduced in dance experts. Related work involving Western, Indian, Japanese, and Iranian contexts further suggests that cultural background, enculturation, and acculturation can shape dance preferences and emotion recognition.^30^^,^^31^^,^^47^ Future research should consider that concepts such as dancing bodies, spatiality, movement quality, aesthetic values, communicative functions, and expertise criteria are often framed through specific cultural lenses and may not apply universally across dance traditions. Deconstructing these ethnocentric biases is essential to place dance forms across different cultures on an equal footing and promote a more inclusive and representative study of dance.

Learning from studies with non-Western, Educated, Industrialized, Rich, and Democratic (WEIRD) dance traditions and participants is critical for generalizing to and learning from other populations.^171^ For example, Western ballet is typically structured around syllabus-based training focused on technique,^86^^,^^87^ whereas Iranian classical dance is more imitation-based, allowing expressive movement at earlier learning stages.^29^^,^^146^ Because dance training style influences aesthetic preferences and the perception of unfamiliar dance styles, exploring embodied concepts such as corporeality and spatiality within different dance contexts could enhance cross-cultural understanding. The complex nature of dance also calls for multidisciplinary and mixed-method approaches to fully capture its dynamic role in human culture.

#### Best practices and recommendations

Both cultural specificity and universal foundations of dance behaviors are important mechanisms to study. Mechanisms such as entrainment and simulation may be shared across humans, yet their manifestation is shaped by cultural context. Participants’ cultural background, prior exposure, and experiences of enculturation or acculturation can be assessed with questionnaires (e.g., the Enculturation and Acculturation Quiz; EAQ^172^). Findings from one dance tradition cannot be generalized to all dance traditions.

### Translational research with dance for health and wellbeing

The benefits of dance practice in the general population are noted across health-related scientific disciplines.^15^^,^^173^^,^^174^^,^^175^^,^^176^ Dance-based interventions draw on partnered (e.g., standard dance, Argentine tango^177^), group (e.g., jazz dance, contemporary, hip-hop^178^), and traditional dance forms (e.g., African dance Nogma^179^). In neurological diseases, dance interventions have supported treatment adherence, mental health, psychosocial functioning, cognitive capacity, and motor skills. In Parkinson’s disease, meta-analyses of randomized controlled trials (RCTs) have found that dance improves motor symptoms, functional mobility, cognition, and quality of life.^180^^,^^181^^,^^182^ Similar benefits have been reported in oncology and cardiac disease populations, where dance supports increased physical activity, mental health, and overall quality of life.^173^ Yet, recent reviews have also reported mixed, small, or contradictory effects in some populations and outcome measures.^183^

Variability in intervention protocols, sample characteristics, and outcome measures continues to challenge the field.^184^ Heterogeneity in study designs, including differences in sample sizes, age groups, treatment frequencies, specification of treatment factors, and clinical populations—and importantly, of dance style—makes it difficult to draw definitive conclusions about efficacy.^185^ For example, Argentine tango has been associated with greater improvement in motor-related symptoms in individuals with Parkinson’s disease compared to other dance forms.^186^ Identifying what differentiates Argentine tango from styles such as Waltz for individuals with Parkinson’s disease may help tailor dance interventions to better target specific neurocognitive, emotional, or motor processes. In general, interventions often lack standardization and valid control groups, further complicating comparisons across studies. Consequently, distinguishing genuine null effects from limitations in the available evidence remains challenging.

A notable exception is dance movement therapy (DMT). DMT is a validated psychotherapeutic dance intervention package with clear therapeutic factors approved for clinical settings. DMT is grounded in the principle that physical movement can reflect emotional states, and that modifying these movement patterns can influence both psychological and social experiences.^187^ A recent scoping review of DMT and other Creative Arts Therapies (CATs) identified therapeutic factors at three levels.^188^ These include common psychotherapy factors such as the therapeutic alliance, emotional regulation, and the creation of a safe environment. They also include factors shared across CATs, such as artistic engagement, symbolic work, self-display, and re-enactment. DMT-specific factors include embodiment, understood as experiencing the body and increasing body awareness, expressing, and sharing emotions non-verbally through movement, using movement metaphors and bodily sensations for insight, and artistic agency. A meta-analysis about DMT showed that expressivity was the treatment factor most directly linked to reductions in symptoms of depression and anxiety.^175^^,^^189^ Relational processes, such as kinesthetic empathy, mirroring, movement dialogue, and synchrony, are also central to DMT. In clinical populations, DMT has been shown to reduce symptoms of depression, anxiety, and stress.^190^ As a psychotherapeutic approach, DMT may offer benefits beyond general physical exercises, meditation, or other CATs, including enhanced proprioception and interoception, refined motor imagery, and improved emotional awareness, capacities also developed through professional dance training.^5^

Dance-based (non-DMT) interventions can use DMT as a model for implementing structured methods and identifying factors that may contribute to positive health outcomes, optimizing dance style choice, treatment duration, frequency, and intensity of any intervention for a specific health outcome.

#### Best practices and recommendations

Standardized practices and methodological improvements are needed across domains of dance-based interventions for health. Priorities include larger sample sizes, rigorously designed RCTs with appropriate blinding, long-term follow-ups, development of disorder-specific protocols,^175^ specification of treatment factors, reporting of key intervention variables (e.g., frequency, duration, facilitator expertise), use of standardized outcome measures, and an operationalization statement about how key variables are hypothesized to modulate the neurocognitive process under investigation, ensuring also gender balance and representation of minoritized and Indigenous groups.^191^ Table 4 proposes a series of characteristics of a dance style that should be reported in intervention studies.

**Table 4:** Characteristics to note about a dance style in an intervention study

Characteristic
Choice of dance style; solo, partner, group
Time spent on technique learning, on choreography practice, on free dancing, on stretching
Is direct eye contact part of the class
Time spent in synchronous movement
Time spent touching another human being (hands, arms)
Types of movements and styles that are part of the curriculum
Focus on choreography and freestyle dance
Involvement of group work, touch and dance-making exercises
Structure of a typical class; session goals
Teaching methods
The learning process – from copying to creation and self-expression
Feedback
Choice of music
Dance program across year groups; curriculum, class content and choreography framework
Number of children in each class
Dance class composition

*Note*: Guide for researchers designing intervention studies. We propose adding such table to the paper and complementing the column ‘the current study’ with the characteristics of the dance intervention in question.

By strengthening the theoretical and methodological foundations of dance-based interventions, future studies can contribute to the development of evidence-based guidelines for healthcare and policy. We recognize that dance-based health research remains underfunded compared with other arts-based therapies, such as music and art therapies, and that high methodological standards can be challenging under limited resources. Yet, even partial alignment with these principles can move the field forward and strengthen its credibility, visibility, funding opportunities, and translational impact.

## Conclusion

By examining what defines dance, how dance expertise can be measured, and how dance can be studied across laboratory and real-world contexts, this seven-principle framework promotes a systematic approach.

Emphasizing inclusivity, individual variation, cross-disciplinary collaborations, replicability, and visibility, the framework supports evidence that can deepen our understanding of dance’s impact on individuals and communities. Ultimately, it aims to bridge academic inquiry and societal application while fostering innovation across disciplines.

## Acknowledgments

The authors would like to thank Professor Anjan Chatterjee, Professor Fredrik Ullén, and Dr. Klaus Frieler for their thoughtful feedback on the manuscript. Further, we would like to thank Felix Bernoully for help with figure preparation for this article.

Funding: This project has received funding from the European Union’s Horizon 2020 research and innovation programme under the Marie Sklodowska-Curie grant agreement no 101031774. J.F.C. was supported by the 10.13039/501100004189Max Planck Society.

## Declaration of interests

The authors declare no competing interests.
